# Off-label drug use during the COVID-19 pandemic in Africa: topic modelling and sentiment analysis of ivermectin in South Africa and Nigeria as a case study

**DOI:** 10.1098/rsif.2023.0200

**Published:** 2023-09-13

**Authors:** Z. Movahedi Nia, N. L. Bragazzi, A. Ahamadi, A. Asgary, B. Mellado, J. Orbinski, L. Seyyed-Kalantari, W. A. Woldegerima, J. Wu, J. D. Kong

**Affiliations:** ^1^ Africa-Canada Artificial Intelligence and Data Innovation Consortium (ACADIC), York University, Toronto, Ontario, Canada; ^2^ Laboratory for Industrial and Applied Mathematics (LIAM), York University, Toronto, Ontario, Canada; ^3^ Advanced Disaster, Emergency and Rapid-response Simulation (ADERSIM), York University, Toronto, Ontario, Canada; ^4^ Dahdaleh Institute for Global Health Research, York University, Toronto, Ontario, Canada; ^5^ Department of Electrical Engineering and Computer Science, York University, Toronto, Ontario, Canada; ^6^ Faculty of Computer Engineering, K.N. Toosi University, Tehran, Iran; ^7^ School of Physics, Institute for Collider Particle Physics, University of the Witwatersrand, Johannesburg, South Africa

**Keywords:** ivermectin, Twitter, topic modelling, emotion analysis, RoBERTa, COVID-19 pandemic

## Abstract

Although rejected by the World Health Organization, the human and even veterinary formulation of ivermectin has widely been used for prevention and treatment of COVID-19. In this work we leverage Twitter to understand the reasons for the drug use from ivermectin supporters, their source of information, their emotions, their gender demographics, and location information, in Nigeria and South Africa. Topic modelling is performed on a Twitter dataset gathered using keywords ‘ivermectin’ and ‘ivm’. A model is fine-tuned on RoBERTa to find the stance of the tweets. Statistical analysis is performed to compare the stance and emotions. Most ivermectin supporters either redistribute conspiracy theories posted by influencers, or refer to flawed studies confirming ivermectin efficacy *in vitro*. Three emotions have the highest intensity, optimism, joy and disgust. The number of anti-ivermectin tweets has a significant positive correlation with vaccination rate. All the provinces in South Africa and most of the provinces of Nigeria are pro-ivermectin and have higher disgust polarity. This work makes the effort to understand public discussions regarding ivermectin during the COVID-19 pandemic to help policy-makers understand the rationale behind its popularity, and inform more targeted policies to discourage self-administration of ivermectin. Moreover, it is a lesson to future outbreaks.

## 1. Background

‘Off-label’ or ‘expanded drug use’ refers to an unapproved use of a drug, that is to say, any use beyond what regulatory agencies have reviewed and authorized to be marketed in a country, as indicated on the product label. Self-administration of unauthorized drugs can pose serious health problems and represents a global public health concern. Self-administration of off-label drugs has become very widespread for treating SARS-CoV-2 during the COVID-19 pandemic [1]. Although several substances such as nirmatrelvir/ritonavir, dexamethasone, remdesivir, and molnupiravir gained approval or conditional approval for mild or severe cases of COVID-19, other medications such as hydroxychloroquine (HCQ), chloroquine (CQ), lopinavir/ritonavir, ruxolitinib, colchicine, doxycycline and ivermectin have been strongly rejected by the World Health Organization (WHO), since randomized controlled trials indicated that they had no clinical efficacy in prevention or treatment of coronavirus, and were rather harmful [2,3]. HCQ/CQ use was found to be associated with a rise in the risk of ventricular arrhythmias and subsequent death of hospitalized patients [2,3].

**Figure 1.**
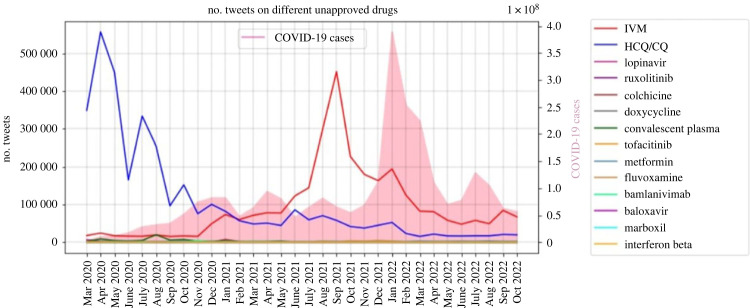
Popularity of unapproved drugs for COVID-19 on Twitter for the whole world.

With the emergence of new SARS-CoV-2 variants, ivermectin gained the most popularity among all the different unapproved drugs. Figure 1 shows the total number of tweets on prohibited drugs used during the COVID-19 pandemic for the whole world. It can be observed that HCQ and CQ were popular at the beginning of the pandemic but lost attention afterwards. However, the volume of the tweets on ivermectin dramatically increased, especially during the third and fourth waves of COVID-19, and it still remains high. Other drugs did not gain that much attention on Twitter during the COVID-19 pandemic.

Ivermectin may have antiviral properties against RNA viruses such as SARS-CoV-2 *in vitro*. However, the dosage required to reach *in vitro* efficacy is very toxic to the human body [4]. Ivermectin toxicity includes symptoms such as ataxia, weakness, decreased consciousness, confusion, hallucinations, gastrointestinal distress, nausea, vomiting, diarrhoea, hypotension, seizure, coma and death [5,6]. Therefore, it was not approved for treatment or prevention of COVID-19. Unfortunately, this did not stop the distribution of the drug through black markets [7,8]. Due to shortages of the human formulation of ivermectin, people even consumed ivermectin intended for livestock [9]. Consequently, during the COVID-19 pandemic, hospitalizations caused by veterinary form and overdosage of human form of ivermectin increased [10]. Moreover, as a result, those who were in need of ivermectin (e.g. for parasites, for their farm animals/pets) found it hard to have access to the drug [11]. Above all, false anecdotes of ivermectin success discouraged people from taking vaccines [12]. In this work, we study social media to understand the reasons that have caused people to believe in ivermectin and their feelings towards it.

People are increasingly using social media platforms to discuss their beliefs, experiences, and opinions. With the lockdown measurements during the COVID-19 pandemic, even more time was spent on social media platforms [13]. Therefore, social media is broadly used in different areas of research, especially the COVID-19 pandemic [14]. Although social media has widely been studied for different aspects of the COVID-19 pandemic such as macroeconomic consequences [15], indicator prediction [16], mental health problems [17], misinformation [18], and vaccine hesitancy [19], few papers have used it to understand mass opinions on ivermectin. Diaz *et al.* [20] have studied ivermectin from a political point of view. Topic modelling and sentiment analysis were performed on tweets related to ivermectin and posted from the United States. The results show that the overall sentiment of the tweets was negative. However, tweets from democrats had more negative polarity compared to tweets from republicans. In [21] a dataset of tweets posted from the United States, the United Kingdom, Canada and India were analysed. Most of the users posting the tweets were health practitioners, scientists/researchers, or executives. Three main topics were identified from the tweets: one of these was ‘Pierre Kory’, an American critical care physician who advocated off-label use of various drugs as treatments for COVID-19, including ivermectin, described as a ‘wonder drug’ with ‘miraculous effectiveness’ against COVID-19. The other two topics were ‘early treatment’, and ‘be brave and keep fighting against COVID19’. Gouveia *et al.* [22] pointed out the high volume of tweets and searches on Google trends regarding ivermectin, HCQ and CQ in Latin American countries. Koss & Bohnet-Joschko [23] found ivermectin to be one of the most discussed supplements used for COVID-19 prophylaxis and treatment, on social media.

In [24] authors found that there was an increase in purchasing ivermectin from December 2020 to January 2021 in the United States and Canada, and this was statistically in line with the number of posts on ivermectin in social media platforms and the number of searches for ivermectin in Google trends. Authors in [25] studied the posts from Peru related to ivermectin on Facebook. Their results show that most of the posts are rumours that support ivermectin efficacy against COVID-19. In [26] Google trends were examined and topic modelling was performed on Facebook posts to understand ivermectin popularity in Romania. They found that ivermectin was a topic of public concern, as the top voices initiating the conversations were not only anti-vaccine influencers, but also mainstream personalities. Of interest, authors in [27] found a negative correlation between the number of ivermectin Google searches and vaccination rates. They also found that the number of searches on ivermectin is higher in locations that have lower vaccination rates. In [28] posts from South Africa were studied to show that social media is a driver to medicine use, despite availability of scientific evidence. The results show that posts related to ivermectin and HCQ efficacy against COVID-19 had positive sentiments, in general. In [29] four medications, namely, HCQ, ivermectin, molnupiravir and remdesivir, were compared using tweets posted from the United States. They concluded that HCQ and ivermectin are highly politicized and under conspiracy theories, hearsay and celebrity effects. Republicans supported ivermectin and HCQ more than democrats. While the general population supported HCQ and ivermectin, people with healthcare backgrounds opposed it.

Although papers mentioned above provide rich information on public opinions regarding ivermectin, none of them have properly studied the reasons ivermectin has gained so much popularity and the general feelings towards it. In this paper, we fill in the gap by performing topic modelling and emotion analysis on tweets related to ivermectin to understand ivermectin-supporters' reasoning and emotions. Additionally, most of the studies in this area have used publicly available datasets. In this research we use Twitter API academic researcher account which guarantees to return all the tweets available for a certain query to study the aspect [30]. Our dataset is freely available from [31]. Our contribution to this work is four-fold:

(1) To understand assertions made for supporting ivermectin, and their sources.
(2) To understand feelings and emotions towards ivermectin being unapproved by WHO and prohibited by responsible health agencies.
(3) To understand the distribution of public opinions and emotions across gender and different locations.
(4) To provide an extensive Twitter dataset on unapproved drug use during the COVID-19 pandemic that future studies could build up on [31].

Natural language processing (NLP) is a machine learning tool for analysing text. Different NLP tasks are performed to conduct this study. Topic modelling is used to comprehend the different discussions related to ivermectin on Twitter. Stance is the state of a tweet being pro-ivermectin, anti-ivermectin or neutral [29]. We train a model based on transformers to find the stance of the tweets. Then, the stances of the tweets in each topic are identified, and the emotions of each stance are realized. Next, the emotions of the stances and topics for men and women, and for different locations in Nigeria and South Africa are studied. Understanding the demography of pro-ivermectin individuals could be useful in messaging campaigns to reduce the harms of off-label drug use. Nigeria and South Africa are the two African countries that were heavily affected by the ivermectin confusion during the COVID-19 pandemic [32,33]. As this project clarifies the rationale behind ivermectin use for COVID-19, it can help decision-makers inform more targeted policies for promoting safe medication use. Moreover, it is a lesson for future epidemics.

## 2. Methods

### 2.1. The dataset

**Figure 2.**
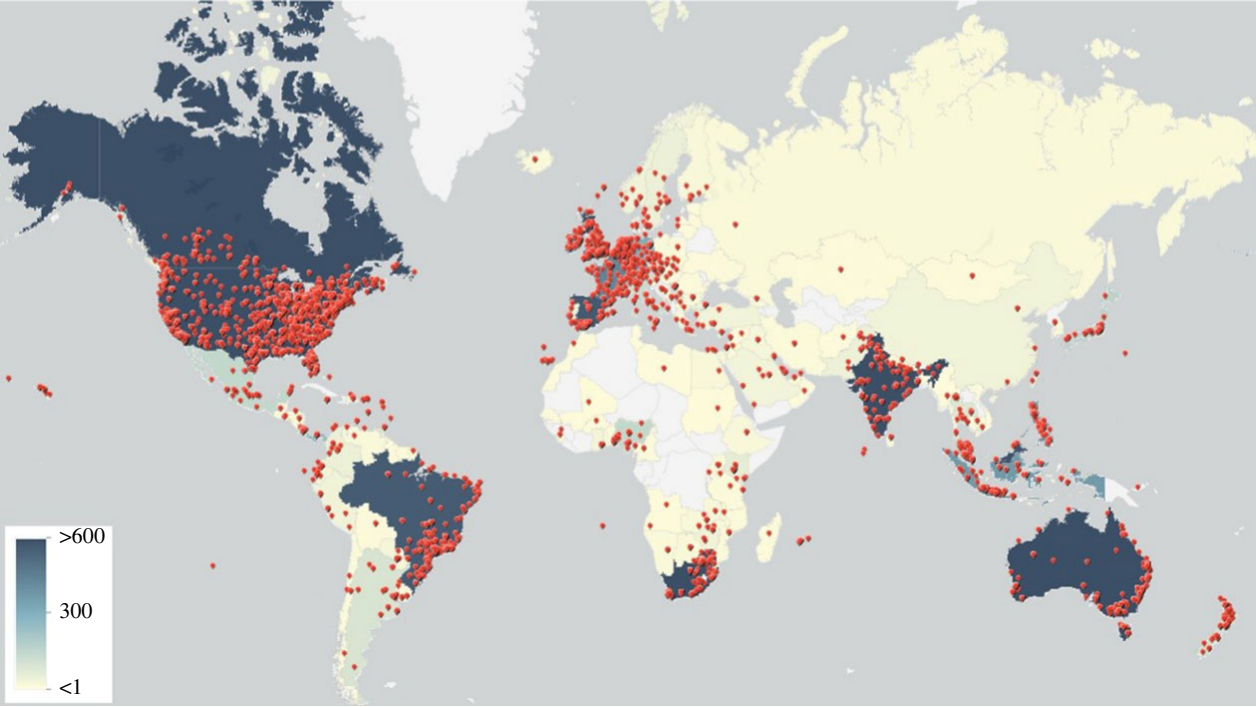
Distribution of geotagged tweets on ivermectin around the globe.

All the tweets, except for retweets, posted from 1 March 2020 to 31 October 2022 with keywords hydroxychloroquine OR hcq OR ivermectin OR ivm OR chloroquine OR lopinavir OR ritonavir OR ruxolitinib OR colchicine OR doxycycline OR ‘convalescent plasma’ OR tofacitinib OR metformin OR fluvoxamine OR lopinavir OR bamlanivimab OR baloxavir OR marboxil OR ‘interferon beta’ are gathered using the full archive search of the Twitter API academic researcher account [30]. The final dataset includes 7 231 116 tweets, and can publicly be accessed from [31]. The tweets containing the keywords ‘ivermectin’ OR ‘ivm’ are extracted. The URLs and mentions (@username) are removed. Since the hashtags carry important information that could help train a better stance detection model, the hashtag terms are preserved and only the # signs are removed for tokenization. After cleaning, null tweets and duplicated tweets are discarded. A number of 3 009 740 tweets are left of which 45 462 are geotagged and visualized using ArcGis online (figure 2) [34].

Since ivermectin has become tightly related to politics and conspiracy theories, it is valuable to know which countries discuss it the most [32]. Figure 1 shows that tweets related to ivermectin come from all over the world; however, they are more concentrated in Europe and North America. In addition, ivermectin is discussed in Africa dominantly in two countries, namely, Nigeria and South Africa. Overall, 102 and 831 of the geotagged tweets belong to Nigeria and South Africa, respectively. Furthermore, the user-specified location of the tweets is used to extract more tweets from Nigeria and South Africa. It is worth mentioning that there is a car company in Nigeria named Innoson Vehicle Manufacturing (IVM) and tweets related to the Nigerian car company are manually removed. Eventually, 1966 and 22 809 English tweets were pulled out for Nigeria and South Africa, respectively. When the geo-city/province tag of a tweet is available, it provides the province of the tweet. However, for non-geotagged tweets, the province is inferred from the user-specified location field. The provinces of 1417 and 15 430 tweets are identified for tweets from Nigeria and South Africa, respectively.

### 2.2. Natural language processing

The Latent Dirichlet Allocation (LDA) tool from sklearn library of Python 3.8 is used to perform topic modelling on the dataset. The best number of topics is the one that maximizes coherence and minimizes the Jaccard distance [35]. Therefore, 11 topics were identified. Tweets belong to each topic with a probability. The probability is used to find the popularity, stance and emotions of each topic.

A model is fine-tuned on RoBERTa to detect the stance of the tweets [36]. Tweets are manually labelled into three classes, pro-ivermectin, anti-ivermectin and neutral. After balancing the labelled dataset, 2841 tweets are left. In order to apply 5-fold cross-validation for evaluating the model, the dataset is divided into five different balanced parts. Five different models are trained and tested, each time a different part is set as the test dataset. Table 1 shows the different metrics of the models trained for detecting the stance of the tweets, and their average as the final evaluation. Table 1 implies that the model recognizes all the three classes very well, since it has a good precision, recall and F1-score for all the three classes of all the five models, and their average. Moreover, the overall accuracy of the model is 73%.

**Table 1.** Evaluation metrics of the trained models for detecting stance.

	class	precision (%)	recall (%)	F1-score (%)	accuracy (%)
fold 1	pro-ivermectin	80	72	76	75
	anti-ivermectin	73	78	75	
	neutral	70	79	74	
fold 2	pro-ivermectin	71	68	69	69
	anti-ivermectin	71	81	75	
	neutral	66	59	62	
fold 3	pro-ivermectin	71	65	67	67
	anti-ivermectin	72	63	68	
	neutral	60	72	65	
fold 4	pro-ivermectin	86	82	78	77
	anti-ivermectin	74	70	77	
	neutral	70	75	72	
fold 5	pro-ivermectin	78	75	77	78
	anti-ivermectin	80	84	82	
	neutral	75	73	74	
final	pro-ivermectin	77	72	73	73
	anti-ivermectin	74	75	75	
	neutral	63	72	69	

Two pretrained emotion analysis models, Pysentimiento [37–39] and Cardiffnlp [40], are used to find the emotions of each topic. Emotion is initially detected using Pysentimiento, which classifies text into 7 classes: joy, surprise, sadness, anger, fear, disgust and other. However, many of the tweets that are classified as other by Pysentimiento are classified as optimism by Cardiffnlp which classifies text into 4 emotions: joy, optimism, anger and sadness. Therefore, optimism was added to the 7 emotion groups of Pysentimiento with the intensity score of ‘optimism’ multiplied by the intensity score of ‘other’.

To find the gender of the users, first genuine users are separated from bots using the source field of the tweets [41]. If the source field of a tweet is ‘Twitter for Android’, ‘Twitter for iPhone’, ‘Twitter for iPad’, ‘Twitter for Mac’, etc., the tweet is posted by a genuine user [41]. Next, the gender of the users are recognized using their profile images and their tweets through the method explained in [42]. To evaluate the accuracy of the emotion-classification model on the dataset, 1102 tweets are manually labelled based on the 8 different emotion classes, i.e. optimism, disgust, joy, fear, anger, sadness, surprise and other. By comparing the labelled dataset with the predicted values, 72% accuracy is found for the emotion analysis model. For the gender classification model, 316 tweets are manually labelled into female and male classes, using their profile images, and names. The accuracy of the gender recognition model is 86%, on the dataset. Table 2 shows the accuracy, precision, recall, and F1-score of the sentiment analysis and the gender recognition models on the labelled datasets.

**Table 2.** Evaluation metrics for the sentiment analysis and gender recognition models.

class	precision (%)	recall (%)	F1-score (%)	accuracy (%)
*sentiment analysis*				
other	63	68	65	72
optimism	73	69	71	
disgust	66	71	68	
joy	76	80	78	
anger	65	68	67	
fear	74	71	73	
sadness	79	76	78	
surprise	83	85	84	
*gender recognition*				
female	79	84	81	86
male	90	88	90	

The genders of the authors of 1289 and 16 117 tweets from Nigeria and South Africa are recognized, respectively. For both countries, the authors of 80% and 20% of the tweets are identified as male and female, respectively. It has been acknowledged in other studies for other countries as well that men participate more than women in ivermectin discussions [29]. The reason could be because ivermectin has become a political concern, and men are more interested than women in politics. Finally, chi-square score, ANOVA and Mann–Whitney *U*-tests are used to compare the different topics, stances, emotions and gender.

## 3. Results

### 3.1. Topic and stance analysis

After carefully studying the 11 different topics, many of the reasons ivermectin believers and disbelievers bring are identified. The topics generally include the following discussions (the results are summarized in table 3):

(1) Topic #1, consuming animal formulation of ivermectin: Tweets in this topic are mostly related to the animal or human formulation of ivermectin. Anti-ivermectin tweets criticize the consumption of the animal form of ivermectin. Some of them refer to ivermectin as horse dewormer or horse paste. Tweets also describe how hospital beds are occupied by patients with ivermectin toxicity symptoms caused by consuming the veterinary formulation. Pro-ivermectin tweets explain that ivermectin is approved for human use as well, and it is safe for COVID-19 treatment. Some even argue that they have used the animal form of ivermectin to cure COVID-19.
(2) Topic #2, waiting for official decision on ivermectin: In this topic anti-ivermectin tweets mostly indicate that one should wait until sufficient evidence proves the ivermectin efficacy. While ivermectin believers dispute that by the time ivermectin is approved the damage is done.
(3) Topic #3, early treatment with ivermectin: In this topic pro-ivermectin tweets vehemently support the idea that ivermectin could eliminate COVID-19 at its early stages, and before the symptoms get severe, while anti-ivermectin tweets deny it and discuss that there is no early treatment for COVID-19.
(4) Topic #4, ivermectin dosage/usage instruction: Pro-ivermectin tweets in this topic mainly recommend the dosage of ivermectin and recommended supplementary ingredients for prevention or treatment of COVID-19. Anti-ivermectin tweets point out that the required dosage of ivermectin for fighting COVID-19 is very toxic to the human body.
(5) Topic #5, ivermectin and big-pharma: Pro-ivermectin tweets claim that vaccines are being promoted and ivermectin is being suppressed because, unlike vaccines, ivermectin is cheap and does not profit the big-pharma. Anti-ivermectin tweets indicate that Merck, the producer of ivermectin, has disapproved the use of ivermectin for COVID-19 as well. However, ivermectin supporters believe that Merck is looking for big money too.
(6) Topic #6, ivermectin is/is not anti-parasite/viral/inflammatory: Anti-ivermectin tweets in this topic explain that ivermectin cannot cure COVID-19 because ivermectin is an anti-parasitic drug, while COVID-19 is a virus. Some pro-ivermectin tweets in this topic reason that ivermectin is anti-inflammatory, so it can alleviate COVID-19 symptoms. Some other pro-ivermectin tweets argue that ivermectin has been found effective against viruses such as malaria, Zika virus, dengue fever, West Nile and even HIV, yet, its efficacy against COVID-19 is rejected.
(7) Topic #7, ivermectin reduces/brings symptoms: In this topic ivermectin supporters claim that ivermectin relieves COVID-19 symptoms and brings milder illness. Ivermectin opposers imply that ivermectin use for COVID is harmful, and many people are being hospitalized due to ivermectin toxicity symptoms.
(8) Topic #8, mass use of ivermectin in some countries: In this topic ivermectin supporters mainly discuss how mass distribution of ivermectin in countries such as India, Australia and Mexico was able to contain COVID-19. However, ivermectin opposers disagree and indicate that not only did ivermectin have no effect on the number of COVID-19 cases in these countries but it was also harmful and increased the number of hospitalizations as well.
(9) Topic #9, clinical trials of ivermectin: Ivermectin believers in this topic refer to the studies that support ivermectin efficacy against COVID-19 and claim that ivermectin kills COVID-19. However, tweets against ivermectin point out that ivermectin kills COVID-19 *in vitro*. Randomized clinical trials suggest that ivermectin is not effective for treating or preventing COVID-19.
(10) Topic #10, ivermectin/vaccines are toxic: In this topic, pro-ivermectin tweets argue that while vaccines are newly produced and not properly tested, ivermectin is a safe drug that has been in use for a long time. Therefore, vaccines are toxic, not ivermectin. Ivermectin is being suppressed, so that vaccines could be approved as an emergency medication. This pandemic is planned to cause fear and force everybody into vaccination. On the other hand, anti-ivermectin tweets indicate that vaccines have been tested and their efficacy has been confirmed. Therefore, vaccines are safe and effective, and ivermectin is toxic and harmful.
(11) Topic #11, news/research on ivermectin: This topic includes articles, reviews, and research published on the efficacy of ivermectin against COVID-19. Ivermectin supporters refer to different studies that confirm the efficacy of ivermectin. Nonetheless, ivermectin opposers point out that those studies are fraud, on a small limited scale, or *in vitro*. Randomized clinical trials deny the efficacy of ivermectin.

**Table 3.** Eleven topics are identified for tweets.

no.	1	2	3	4	5	6	7	8	9	10	11
keywords	horse, cow, pet, cattle, animal, human, veterinary, dewormer	evidence, wait, enough, sufficient, decision, scientific, recommend	early, treatment, success, fight	high, dose, vitamin, zinc, aspirin, pregnant, prevent, prophylaxis	cheap, big, pharma, money, government, profit	parasite, dewormer, viral, virus, inflammatory	symptom, mild, severe, hospital, hospitalize	India, Mexico, Australia, Japan, Slovakia, Zimbabwe, cases, control, rate	vitro, clinical, trial, random, kill unapproved	test, harm, toxic, emergency, approval, fear, planned	study, research, review, meta, speak, scientist, data, read, fraud, article, news
examples for ivermectin supporters	• ivermectin is used for humans as well not only for horses. Quit the media bias	• my dad died from COVID-19 while waiting for ivermectin application approval	• u've ignored early treatment and the importance of it in this fight for the whole period. Pls don't shift blame or try and pin this on others. 250K South Africans are dead cause of ur push to ignore early treatment. Ur voice was silent on IVM *et al.* cause ur agenda was vax	• I have used ivermectin and other supportive therapies like zinc vitaminD vitaminC successfully. It was demonized because it makes no profit for big pharma and their medical salespeople 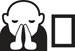	• big pharma are no longer going to make big money from vaccine. IVERMECTIN is cheap	• in addition to anti-viral and anti-inflammatory actions, ivermectin also has potential to reduce coagulation making it ideal for COVID-19	• ivermectin patients recovered 2 days quicker from mild symptoms, and they found that insignificant 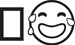	• where ivermectin has been used numbers are down, no hospital cases, no vaccine. Like India, Mexico, Slovakia, now Japan has called for it! Explain that	• there are many studies go and read this one published by Lancet. The effect of early treatment with ivermectin on viral load, symptoms and humoral response in patients with non-severe COVID-19: a pilot, double-blind, placebo-controlled, randomized clinical trial	• ivermectin tablets and /or fenofibrate tablets are the best way to prevent and cure COVID19 and variants. The toxic spike protein jab is more harmful and fatal than COVID19 and variants	• when fraudulent and potentially flawed studies are excluded from the meta-analysis, ivermectin's benefit disappears
	• you can get ivermectin in a paste form at animal feed stores. I mean sounds crazy, but I've known people to use it. Particularly two ppl who had Covid used it and was 100% 3 days later	• off-label prescription ivermectin has sufficient evidence of efficacy. While waiting for vaccination		• have an ivermectin 18mg tablet once a day only a week for 8 weeks plus 150mg aspirin a day for 30 days COVID19 70kg non pregnant adult	• there's no money to be made, or significantly less, from ivermectin. Big pharma will bully our inept little government		• no doubt she is unvaxxed like all Dems and takes ivermectin, so you would expect mild symptoms	• go look at Israel and India, India uses ivermectin to treat COVID and it's almost non existent over there while in Israel, most of the population is double jabbed but yet cases and hospitilizations are rising	• ivermectin haters don't want to see thousands of lives saved. They only want to see randomized trials	• the same with ivermectin. They couldn't get emergency use approval for vaccines if there were other therapeutic treatments	
	• ivermectin, if you cannot get pills for humans from a pharmacy, try your friendly vet or farmer that uses it for their sheep, cows or horses. Ivermax, Ivomec, etc. many brand names. Check the safe dosage !									• they are storming pharmacies to take the ivermectin due to a lack of studies yet they push a vaccine on us that has only be tested for 6 months	
examples for ivermectin opposers	• you are not a horse. You are not a cow. Seriously, y'all. Stop it	• ivermectin is still hugely controversial as treatment for COVID-19. No consensus yet. Prefer to wait until there is	• high-dose ivermectin for early treatment of COVID-19 (COVER study): a gold standard recent clinical study again showing it doesn't work for COVID-19	• another cocktail—ivermectin, dexamethasone, enoxaparin and aspirin. And as I've pointed out elsewhere, ‘ivermection used in a higher dose than approved for parasitosis’. Untested! A few other things I'd like to take a look at incl CFR	• big pharma developed ivermectin that Rogan keeps hawking. But sure you're the smart one	• because IVM is an anti-parasatic. Covid is a virus. An anti-parasatic does not magically become an anti-viral	• ivermectin has been touted as a wonder drug for COVID-19, but a new trial in Colombia revealed that it did not alleviate mild symptoms in infected people	• any studies to report on from India? Cases and deaths out of control and people swallow ivermectin by the thousands	• there are literally randomized, double blinded clinical trials that show that not only does IVM do nothing again covid but that it actually worsens your clinical outcomes in hospital	• true, so why is emergency use of IVM OK, but roll out of a tested vaccine not	• when fraudulent and potentially flawed studies are excluded from the meta-analysis, ivermectin's benefit disappears
	• how hard COVID-19 antivaxxers claim I say ivermectin is ONLY a horse dewormer. I don't. I do say it is used to deworm horses, prevent river blindness in humans, is an antiparasitic. Those are facts. Antivaxxers are allergic to facts				• the big pharma company that makes ivermectin (and would make $billions if it did) says it does nt cure covid, but that's not enough for some people		• popular drug does not alleviate mild COVID-19 symptoms, study finds	• I totally agree. If after the disaster in India anyone suggests ivermectin or chloroquine works and cuts cases, please get some help. The odds no longer stack up, the massive infection and death rate are suggesting something different	• I totally agree. If after the disaster in India anyone suggests ivermectin or chloroquine works and cuts cases, please get some help. The odds no longer stack up, the massive infection and death rate are suggesting something different	ivermectin substances not mentioned on packaging could be harmful	
										maybe he should go to SAHPRA to oversee the approval of the import of ivermectin or to at least ensure they are hastily testing it as a medicine that Covid 19 can be treated with. To visit overcapacitated hospitals will not save lives	
discussion in general	consuming animal formulation of ivermectin	waiting for official decision on ivermectin	early treatment with ivermectin	ivermectin dosage/usage instruction	ivermectin and big pharma	ivermectin is/is not anti-parasite/viral/inflammatory	ivermectin reduces/brings symptoms	mass use of ivermectin in some countries	clinical trials of ivermectin	ivermectin/vaccines are toxic	news/research on ivermectin

**Figure 3.**
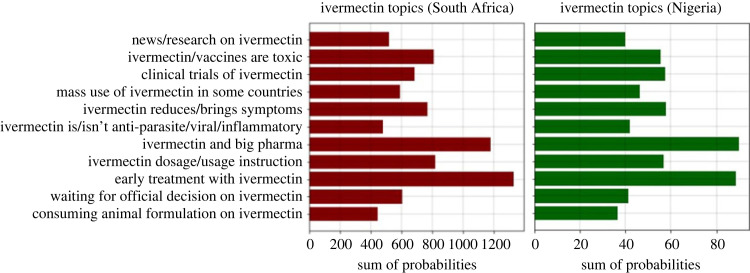
Popularity of each topic in Nigeria and South Africa.

**Figure 4.**
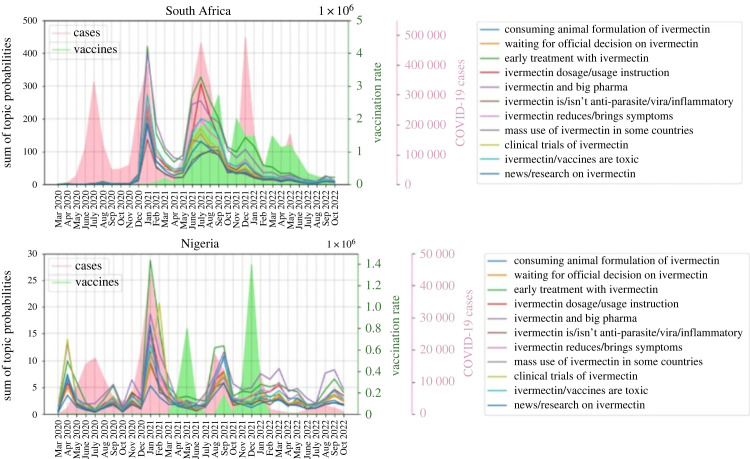
Popularity of different topics over time for Nigeria and South Africa.

Figures 3 and 4 show that two topics, ‘early treatment with ivermectin’ and ‘ivermectin and big pharma’ have the highest popularity in both countries. Figure 3 shows the popularity of each topic. A tweet is related to each topic with a probability. By adding the probabilities of a topic over all the tweets, the popularity of that topic is obtained. Figure 3 shows that topic popularities have a similar distribution in South Africa and Nigeria. There values are 97% correlated with *p*-value of 3.43 × 10^−7^. Figure 4 shows the popularity of different topics over time for Nigeria and South Africa.

**Figure 5.**
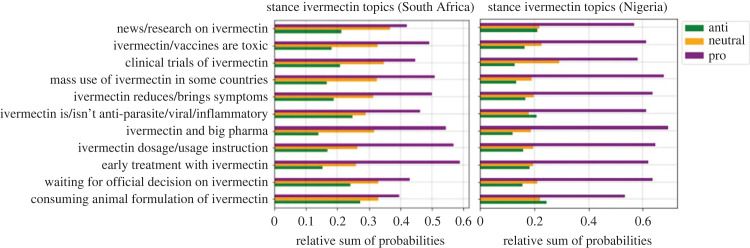
Stance of different topics for Nigeria and South Africa.

Figure 5 shows the stance of each topic as a percentage. In both countries three topics, ‘consuming animal formulation of ivermectin’, ‘ivermectin is/isn't anti-parasite/viral/inflammatory’, and ‘news/research on ivermectin’, have a higher anti-ivermectin percentage compared to other topics. Moreover, in both countries, five topics, ‘early treatment with ivermectin’, ‘ivermectin dosage/usage instruction’, ‘ivermectin and big pharma’, ‘ivermectin reduces/brings symptoms', and ‘mass use of ivermectin in some countries’, have a higher pro-ivermectin percentage compared to other topics. In general, topics in Nigeria have a higher pro-ivermectin percentage compared to South Africa, and topics in South Africa have a higher neutral percentage compared to Nigeria. The chi-square test *p*-value of 0.0086 also confirms that the stance distribution on topics is very different for the two countries.

**Figure 6.**
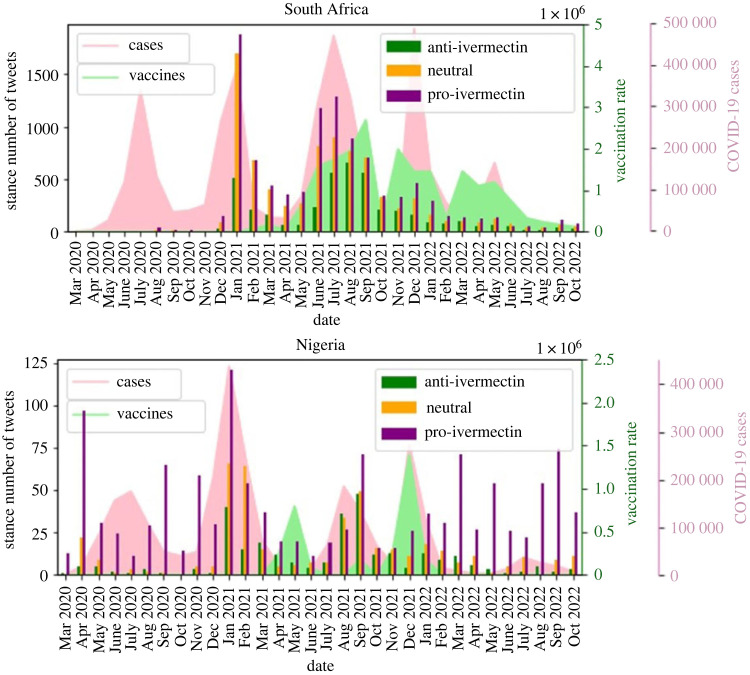
Popularity of each stance over time.

Figure supplementary_figure_1.pdf in the electronic supplementary material shows a word cloud for each stance, after ‘ivm’ and ‘ivermectin’ are removed from the tweets. Word clouds for anti-ivermectin, neutral and pro-ivermectin tweets include their respective keywords (e.g. horse, animal, human, and evidence for anti-ivermectin, clinical, trial and studies for neutral, and effective, early, treatment, kill for pro-ivermectin). Interestingly, all of the figures include the word ‘vaccine’ which implies the debate between ivermectin and vaccines on Twitter. This debate is also confirmed by research that studies COVID-19 vaccination dynamics using social media [43]. Figure 6 shows the relative stance of the tweets for each country over time. The pink and green diagrams are the COVID-19 infection and vaccination rates respectively. Figure 6 shows that in South Africa over time, especially after June 2021 the number of anti-ivermectin tweets increased. The reason could be the vaccine roll-out discouraging people from using unapproved medication. Chi-square test *p*-values for relative stance from March 2020 to June 2021 compared to July 2021 to October 2022 is 1.94 × 10^−23^, meaning that the stance distributions on the two time sets are very different from each other. For Nigeria, from 5 June 2021 to 13 January 2022, Twitter was banned. Figure 6 shows that the tweets had a higher neutral and anti-ivermectin portion in that period. After removing that period from the dates, the chi-square *p*-value (6.93 × 10^−52^) still shows that the stances of the tweets before and after vaccinations are very different from each other.

Table 4 shows the correlation between the number of COVID-19 cases and tweets on ivermectin, and the correlation between the number of vaccinations and different stances for South Africa and Nigeria after removing the banned period. Table 3 shows that discussions on ivermectin increase on Twitter during the COVID-19 waves. Moreover, the number of anti-ivermectin tweets has a strong positive correlation with the number of administered vaccines, and the numbers of pro-ivermectin and neutral tweets have a weak negative or insignificant correlation with the number of administered vaccines. It could be concluded that with vaccine rollout people become more anti-ivermectin and consume the drug less.

**Table 4.** Correlation between number of cases and total number of tweets on ivermectin, and correlation between different stances and number of administered vaccines for South Africa and Nigeria after removing the Twitter-banned period.

Nigeria			South Africa		
	correlation with number of cases	*p*-value		correlation with number of cases	*p*-value
total tweets	0.6094	0.00094	total tweets	0.62784	0.00011
	*correlation with vaccination rate*	*p-value*		*correlation with vaccination rate*	*p-value*
anti-ivermectin	0.4760	0.01396	anti-ivermectin	0.66016	3.87 × 10^−5^
pro-ivermectin	−0.2912	0.14882	pro-ivermectin	−0.34718	0.05154
neutral	0.08968	0.66304	neutral	−0.20192	0.26774

### 3.2. Emotion analysis

**Figure 7.**
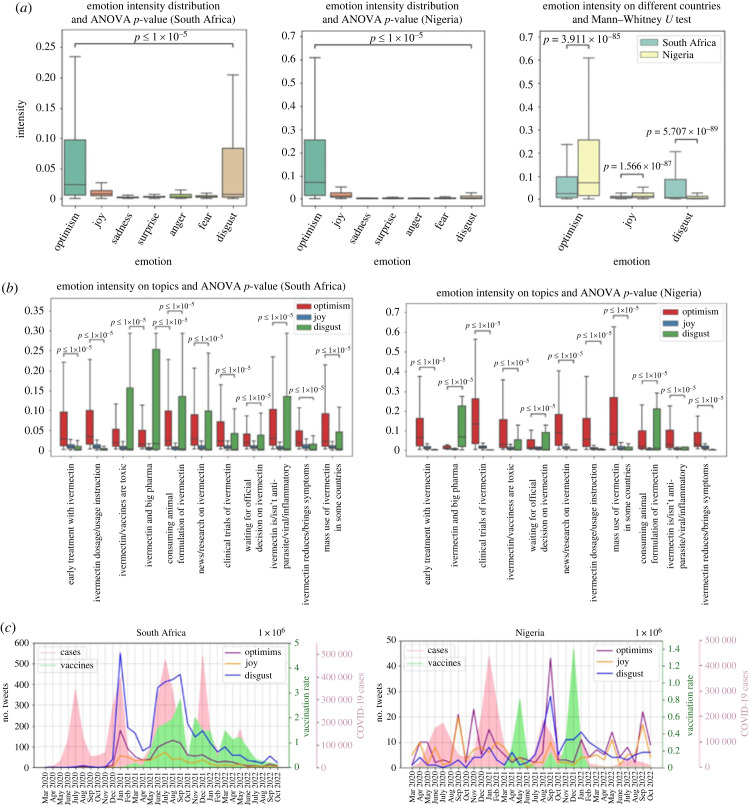
(*a*) Comparing emotions between Nigeria and South Africa, (*b*) comparing emotions of different topics in Nigeria and South Africa and (*c*) emotion over time.

Figure 7*a* shows that three emotions have the highest intensity for different tweets, disgust, joy and optimism (extremely low ANOVA *p*-value). In addition, disgust, joy and optimism has been compared using Mann–Whitney *U*-test between South Africa and Nigeria. The results show that optimism and joy have a higher intensity in Nigeria and disgust has a higher intensity in South Africa. Generally, people in Nigeria are more positive than in South Africa.

Figure 7*b* shows the emotion intensities on different topics. Some topics such as ‘ivermectin/vaccines are toxic’, ‘ivermectin and big pharma’, ‘consuming animal formulation of ivermectin’, and ‘ivermectin is/isn't anti-parasite/viral/inflammatory’ have a really high disgust intensity. On the other hand, topics such as ‘early treatment with ivermectin’, ‘ivermectin dosage/usage instruction’, and ‘mass use of ivermectin in some countries' have a high optimism intensity. Figure 7*c* shows the emotions over time for Nigeria and South Africa. In South Africa the number of tweets for disgust has always been higher than the number of tweets with optimism or joy. However, in Nigeria, mostly the number of tweets with optimism and joy are higher than disgust.

Notably, in figure 7*c* for Nigeria, there is a peak for both optimism and disgust emotions from August to October 2021. By going through the tweets in this period, we learned that they are mostly anti-ivermectin, discouraging unapproved drugs, and promoting vaccines. This observation which is also confirmed by figure 6, could be the effect of vaccine rollouts which happened right before this period. This again shows that providing real medication such as vaccines to people stops them from consuming off-label drugs such as ivermectin. Moreover, papers that study COVID-19 vaccination from a social media perspective confirm that as vaccine supplies become available and rollouts take place, sentiments and emotions towards vaccination level up and become more positive [19,44–46].

### 3.3. Gender and location

**Figure 8.**
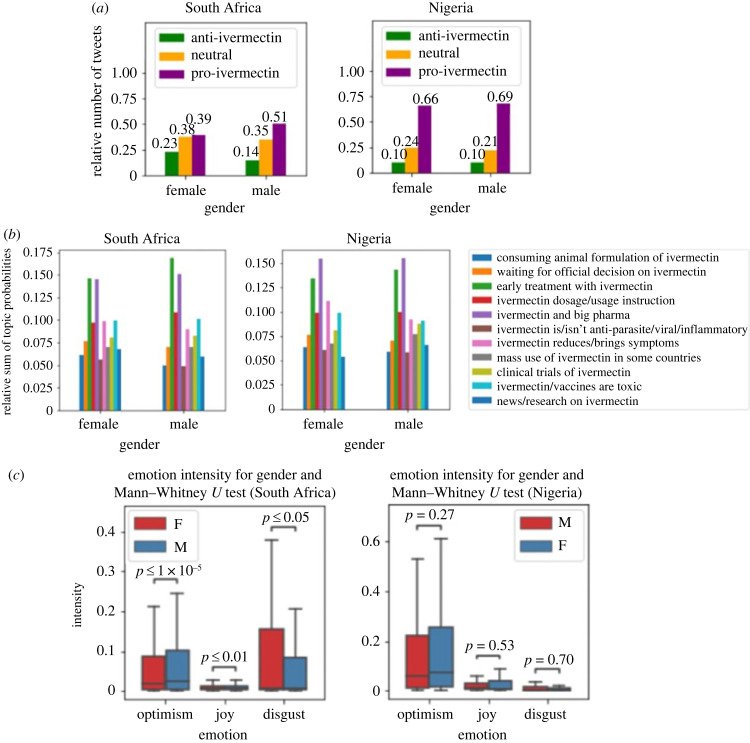
(*a*) Stance distribution on gender, (*b*) topic distribution on gender, (*c*) emotion intensity on gender.

Figure 8*a* shows the distribution of stance on gender. In both countries men are more pro-ivermectin. In South Africa women are more neutral and anti-ivermectin. In Nigeria women are more neutral. Chi-square *p*-value of 0.006 shows that the distribution of stance on gender is very different for the two countries. The reason for this result, which is in line with other studies in other countries, may be that men are more concerned with politics compared to women [29]. Figure 8*b* shows gender for different topics. The correlations between topic popularities of men and women for Nigeria and South Africa are 0.95 (*p*-value = 3.31 × 10^−6^) and 0.98 (*p*-value = 2.23 × 10^−8^), respectively. The chi-square *p*-value between the two countries is not significant (0.63) meaning that topic has a similar distribution on gender for the two countries. Figure 8*c* shows the distribution of emotion intensity on gender. Mann–Whitney *U*-test shows that emotions in Nigeria are very similar for men and women, but in South Africa are very different. In South Africa, women have more negative (disgust) emotions and men have more positive (optimism and joy) emotions. Figure 8*c* shows that in Nigeria the intensity of optimism and joy is slightly (but not significantly) higher for women, and the intensity of disgust is slightly (but not significantly) higher for men.

**Figure 9.**
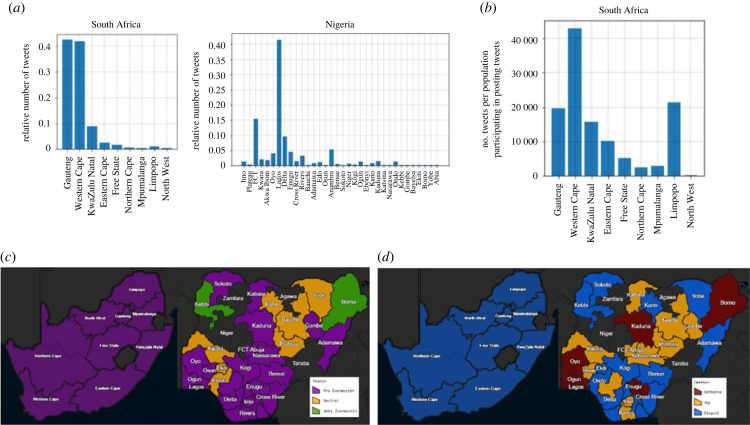
(*a*) The number of tweets for different provinces of Nigeria and South Africa, (*b*) number of tweets per population participating in posting tweets in South Africa, (*c*) stance for different provinces of Nigeria and South Africa, (*d*) emotion for different provinces of Nigeria and South Africa.

Figure 9*a* shows the number of tweets for different provinces of Nigeria and South Africa. Tweets are mostly from Lagos and FCT in Nigeria, and from Gauteng and Western Cape in South Africa. The percentage of Twitter users from each province of South Africa is derived from [47]. The number of tweets from each province is divided by the percentage of Twitter users from that province (figure 9*b*). Figure 9*b* shows that people from Western Cape that use Twitter are more interested in ivermectin compared to other provinces. Figure 9*c* shows that all the provinces of South Africa and most of the provinces of Nigeria are pro-ivermectin. Moreover, figure 9*d* shows that the dominant emotion in all the provinces of South Africa is disgust. However, tweets from Nigeria have more positive emotions, and the dominant emotion in some provinces is optimism or joy.

## 4. Discussion

Social media is increasingly being used for sharing thoughts and opinions. Although posts retrieved from social media are not equally portioned over various age groups, languages, occupations, and locations, previous studies have shown that they provide enough information to understand and analyse mass opinions and compare discussions among different places and demographics [48]. Using NLP tools such as topic modelling, stance analysis and emotion analysis, we were able to identify the reasons behind self-administration of ivermectin for COVID-19.

In general, we classify the source of pro-ivermectin discussions into three groups. The first group refers to the flawed published studies that confirm the efficacy of ivermectin against COVID-19. The increasing number of COVID-19 cases and mortalities and the absence of medical vaccines urged the adoption of repurposed medicine such as ivermectin, despite lack of sufficient evidence [28,49]. However, more robust clinical trials rejected the effectiveness of such medications, later on. Yet, ivermectin is still being promoted as a miracle cure for COVID-19 on social media. Discussions in this group such as ‘early treatment with ivermectin’ and ‘ivermectin reduces symptoms' are mostly optimistic, and hope that the wonder drug ivermectin can end the pandemic. This is a great lesson to future pandemics and emerging diseases. Strict surveillance is crucial for the early research and studies in an epidemic.

The second group exemplifies countries that have approved ivermectin for mass use against COVID-19 such as India, Mexico and Australia as a success story of ivermectin, regardless of how dangerous and life-threatening it could be [50]. These tweets also dominantly have joy and optimism emotions, hoping that ivermectin could change the course of the pandemic.

The third group is the result of conspiratorial quotes narrated by influencers. Ivermectin conspiracy theories such as ‘ivermectin is rejected for emergency approval of the vaccines’, ‘this pandemic is planned to cause fear and enforce vaccines, that is why ivermectin cannot be approved’, and ‘ivermectin is cheap unlike vaccines and does not profit big pharma even the manufacturing company, Merck’ are highly reflected on Twitter [51]. They are discussed with intense disgust. In addition, historical medical abuse in Africa (e.g. Pfizer Trovan tests in Kano, Nigeria in the 1990s [52]) has exacerbated the situation, and escalated scepticism. In response, stricter control and monitoring of social media was informed by authorities. This increased distrust and suspicion even more, and resulted in more propagation of disinformation. Unfortunately, as a result, accessing true and accurate information became more difficult for those who seek for it [28]. Therefore, there is an urgent need to propagate true information. Pharmacists could play an important role in this context as they are the first place people encounter for accessing medicine [28]. Finally, our results show that vaccination rate has a significant positive correlation with the number of anti-ivermectin tweets. This indicates that one major reason for administering ivermectin is not having access to true medication such as vaccines. As a result, promoting vaccines discourages people from consuming ivermectin for treatment or prevention of COVID-19.

## 5. Limitations

Similar to all other social media-based research, this work faces inevitable limitations. A small percentage of people use Twitter. Users are mostly from ages 18 to 49 years old [53]. We were able to analyse only English tweets. Finally, Twitter was banned in Nigeria from 5 June 2021 to 13 January 2022, which is exactly the period that ivermectin had the highest popularity among people and on social media. Yet, a reasonable volume of tweets related to ivermectin was gathered in that period from Nigeria.

## 6. Conclusion and future work

In this work, tweets from South Africa and Nigeria are studied to understand public opinions towards ivermectin, one of the prohibited, yet still widely used drugs for prevention and treatment of COVID-19. The results show that most of the tweets of both countries are pro-ivermectin. There is a debate in social media between ivermectin and vaccines. Anti-vaccine users are mostly pro-ivermectin and vice versa. Two topics are discussed the most on Twitter, ‘early treatment with ivermectin’ and ‘ivermectin and big pharma’. Three emotions have the highest intensity in both countries, optimism, joy and disgust. The emotion in all the provinces of South Africa is dominantly disgust, and the stance is pro-ivermectin. In Nigeria, most provinces are pro-ivermectin, and the emotions in different provinces are more positive than in South Africa. Similar to other works that have been conducted for the USA, we have found that sentiments in Nigeria are not significantly different between men and women [29]. However, in South Africa the intensity of disgust is significantly higher for women, and the intensity of joy and optimism is significantly higher for men. The reason for this must be investigated from diverse perspectives including politics, psychology, culture and lifestyle, and history of self-medication. Since this is out of the scope of our paper, we leave it to future work to build up on our results and dataset and dive deeper into demographics.

The results of this work could be beneficial to policy-makers in informing more targeted policies towards stopping self-administration of unapproved drugs such as ivermectin. Furthermore, it is a lesson to future pandemics.

## Data Availability

Due to Twitter's privacy policy [48], only tweet-ids and user-ids could be shared. The datasets used and/or analysed during the current study are available from [31] with regards to Twitter's privacy policy. The data are provided in electronic supplementary material [54].
